# ETS1-Driven Nucleolar Stress Orchestrates OLR1^+^ Macrophage Crosstalk to Sustain Immunosuppressive Microenvironment in Clear Cell Renal Cell Carcinoma

**DOI:** 10.1155/humu/8856239

**Published:** 2025-10-03

**Authors:** Lei Xiao, Zicheng Zhang, Tong Li, Yuyin Jiang, Yuanxin Liu, Jia Wang, Wei Tang

**Affiliations:** ^1^Department of Endocrinology, Geriatric Hospital of Nanjing Medical University, Nanjing, Jiangsu, China; ^2^Hohai University Hospital, Nanjing, Jiangsu, China

**Keywords:** clear cell renal cell carcinoma, ETS1, immunosuppressive microenvironment, nucleolar stress, OLR1^+^ macrophages

## Abstract

While hypoxia-driven nucleolar stress (NS) has been recognized as a critical modulator of the immunosuppressive tumor microenvironment in clear cell renal cell carcinoma (ccRCC), its mechanistic contribution to disease progression remains poorly defined. To address this gap, we systematically mapped NS-associated molecular landscapes through integrated spatial transcriptomics and single-cell RNA sequencing of ccRCC specimens. Our analysis stratified tumors into two distinct NS subtypes, revealing that high-NS tumors exhibit aggressive clinical behavior, elevated expression of immunosuppressive checkpoints, and significantly reduced survival. At single-cell resolution, high-NS malignant cells displayed enhanced proliferative activity, glycolytic metabolic reprograming, and marked chromosomal instability. Mechanistic investigations demonstrated that hypoxia-induced ETS1 activation orchestrates NS via the MYC/NPM1/DDX17 signaling axis, directly promoting tumor proliferation and metabolic adaptation in preclinical models. Spatial multiomics further uncovered coordinated niche formation between high-NS cells and OLR1^+^ macrophages, with ligand–receptor profiling identifying the EDN1–EDNRA–OLR1 axis as a central mediator of this immunosuppressive crosstalk. Functional validation in syngeneic mouse models confirmed that ETS1 overexpression accelerates tumor growth while enriching OLR1^+^ macrophages with immunosuppressive phenotypes. Clinically, high OLR1^+^ macrophage infiltration correlated with shorter survival across independent cohorts. These findings establish a hypoxia–ETS1–NS–macrophage axis as a key mechanism sustaining ccRCC progression and highlight actionable targets for disrupting protumorigenic immune niches through modulation of the NS pathway.

## 1. Introduction

Clear cell renal cell carcinoma (ccRCC) is a malignant urological tumor originating from the renal tubular epithelium and accounts for approximately 75% of all kidney cancers. Compared with other subtypes, ccRCC exhibits higher invasive potential and a worse prognosis. In 2020, approximately 431,000 new ccRCC cases and 179,000 deaths were reported globally [1, 2]. In recent years, an improved understanding of ccRCC pathogenesis has led to considerable progress in targeted therapy and immunotherapy. Drugs targeting vascular endothelial growth factor and its receptors have been approved for the treatment of ccRCC, and immunotherapy based on immune checkpoint inhibitors, as well as novel combination therapies involving immune checkpoint inhibitors and targeted agents, is being widely investigated [3–5]. However, due to the high heterogeneity of RCC tumors and their immunosuppressive microenvironment, ccRCC patients often show suboptimal responses to these treatments and experience limited survival benefits [6, 7]. This may be attributed to the inherently immunosuppressive nature of the ccRCC tumor microenvironment (TME).

Hypoxia is a common feature of solid tumors and has been strongly associated with the progression of renal cancer [8]. Hypoxic conditions activate hypoxia-inducible factors (HIFs), particularly hypoxia-inducible factor-1 (HIF-1*α*) and HIF-2*α*, which contribute to the formation of an immunosuppressive microenvironment intricately linked to malignant proliferation, angiogenesis, invasion, and metastasis [8–12]. The interplay between tumor cells and immune cells within this hypoxic microenvironment plays a pivotal role in driving tumor progression [13]. In particular, infiltrating macrophages promote immune evasion and therapeutic resistance by releasing various cytokines and chemokines [14]. Recent studies have shown that tumor-derived exosomes can promote M2 macrophage polarization under hypoxic conditions by activating the PI3K–AKT signaling pathway, thereby facilitating the progression of renal cancer [15].

Most notably, studies have shown that hypoxia can induce nucleolar stress (NS) [16]. NS is a cellular stress response triggered by interference in the ribosome biogenesis process. The excessive accumulation of ribosomal proteins caused by NS is closely associated with tumor cell metabolic reprogramming, immune evasion, and treatment resistance [17]. The interaction between tumor cells undergoing NS and the hypoxic, immunosuppressive TME during ccRCC progression has not been extensively explored. Therefore, spatial transcriptomics and single-cell transcriptomics were integrated to map the pathological landscape of ccRCC and to better characterize the occurrence of NS and its contribution to the formation of an immunosuppressive TME.

Our study provides a comprehensive overview of NS in tumor cells under hypoxic conditions within the pathological microenvironment of ccRCC. It further defines molecular subtypes associated with NS and identifies key regulatory pathways in high-NS subgroups that exhibit strong invasive potential. The spatial colocalization of high-NS tumor cells and OLR1^+^ macrophages is closely linked to immunosuppressive phenotypes and may contribute to reduced immunotherapy efficacy. These key regulatory factors are associated with patient prognosis and may represent actionable targets for the treatment of ccRCC.

## 2. Methods

### 2.1. Data Acquisition

A publicly available TCGA-ccRCC dataset was acquired from The Cancer Genomic Atlas (TCGA, https://portal.gdc.cancer.gov/). The scRNA-seq dataset GSE178481 was retrieved from the GEO database (https://www.ncbi.nlm.nih.gov/geo/query/acc.cgi?acc=GSE178481), including eight normal samples and 13 tumor samples. After quality control, a total of 128,179 cells were screened for downstream analyses. Spatial transcriptome sequencing data for ccRCC tissues was acquired from GSE175540 (https://www.ncbi.nlm.nih.gov/geo/query/acc.cgi?acc=GSE175540).

### 2.2. Consensus Clustering

The ConsensusClusterPlus R package, Version 1.60.0, was employed to execute consensus clustering for the purpose of identifying molecular subtypes linked to the NS-associated signature. Subsequently, we evaluated the number of clusters ranging from *k* = 2 to *k* = 9 and repeated that process 1000 times to ensure the robustness and stability of our results. The pheatmap R package, Version 1.0.12, was then employed to visualize the cluster map.

### 2.3. Differential Gene Expression Analysis

The differential expression of mRNAs was evaluated using the limma package (v3.52.2). The screening criteria for differentially expressed mRNAs were set at a significance level of adjusted *p* < 0.05 and an absolute log2 fold-change of ≥ 1 (|log2(fold change (FC))| ≥ 1).

### 2.4. Function Enrichment Analysis

A KEGG enrichment analysis and gene set enrichment analysis (GSEA) of differentially expressed gene sets were performed using the R package clusterProfiler (v4.2.2). Signaling pathways with an adjusted *p* value < 0.05 (after false discovery rate (FDR) correction) were classified as activated pathways and were visually represented using the R package GseaVis (v0.0.1).

### 2.5. Immune Infiltration Analysis

First, the MCP-counter algorithm (v1.2.0) was used to infer the abundance of 10 immune cell types, and differences were assessed using the Wilcoxon rank-sum test. Next, to further identify the immune characteristics, we used cibersortx (https://cibersortx.stanford.edu/) to determine the relative percentages of 22 immune cell types. Subsequently, we compared the relative percentages of the 22 distinct immune cell types in the high-NS and low-NS subgroups.

### 2.6. Prediction of Response to Immunotherapy

A comprehensive tumor immune dysfunction and exclusion (TIDE) analysis was performed to ascertain the response to immunotherapy. We used the TIDE platform (accessible at http://tide.dfci.harvard.edu/) to predict the immunotherapy response by analyzing three key signatures: T cell dysfunction, T cell exclusion, and myeloid-derived suppressor cells (MDSCs), respectively.

### 2.7. Somatic Mutation Analysis

Somatic mutation data for the ccRCC samples were obtained from the TCGA GDC Data Portal in “maf” format. We screened the Top 30 mutated genes and then created waterfall plots using the maftools (v2.12.0) R package to visualize and summarize the mutated genes.

### 2.8. Single-Cell Transcriptome Analysis and Differential Expression Analysis

The Seurat package (v4.1.1) was utilized for an in-depth downstream analysis. Cells that exhibited fewer than 1000 unique molecular identifiers (UMIs) or had > 15% of their genes originating from mitochondria were excluded from the analysis to ensure data quality. To mitigate batch effects and prevent any undue influence from individual patient characteristics on the analysis, the Harmony algorithm was implemented to effectively remove batch effects within the single-cell data. To facilitate dimensionality reduction and the identification of cell subtypes, the FindVariableFeatures function was employed to select the 4000 most variable genes, which were then scaled appropriately. A principal component analysis (PCA) was subsequently conducted using those variable genes. By utilizing the FindNeighbors function, nearest neighbors for graph clustering were determined based on the Top 50 principal components. The FindClusters function was then used to obtain the resulting clusters, and cell visualization was achieved by applying the uniform manifold approximation and projection (UMAP) algorithm. Additionally, gene signatures specific for various cell types were scored within the identified clusters. A differential gene expression analysis between clusters was performed using the “FindAllMarkers” function, with parameters set to min.pct = 0.2, logfc.threshold = 0.2, and only.pos = TRUE. The Wilcoxon's rank-sum test, coupled with the Benjamini–Hochberg method, was utilized to determine *p* values for the comparisons, thereby ensuring robust statistical significance.

### 2.9. Gene Set Level Analysis for scRNA-seq Data

Single-cell signature scoring was executed utilizing the RunAUCell function available in the pochi R package (v0.1.0). To evaluate the differential enrichment of the signature scores across various groups, a two-sided Wilcoxon rank-sum test was performed, which included the Benjamini–Hochberg FDR correction.

### 2.10. Trajectory Analysis

The scTour tool was used to perform a robust inference and prediction of malignant subtype dynamics based on deep learning architecture, and we then analyzed the correlation between pseudotime inferred by the scTour tool and various pathways.

### 2.11. Metabolic Activity Analysis

The R package scMetabolism (v0.2.1) utilizes the AUCell method to evaluate KEGG metabolic pathways, thereby enabling the quantification of metabolic activity within single cells based on the expression matrix derived from scRNA-seq data. We used the singleseqgset (v0.1.2) package to quantify differences of enrichment between metabolic pathways. The mean gene expression level was calculated, and the log two FC between the specific cell type and the other cells was determined.

### 2.12. Transcription Factor (TF) Regulon Analysis

The pySCENIC package was used to conduct a comprehensive analysis of the regulatory network and regulon activity. We then used that analysis to identify subgroup-specific TFs via the Wilcoxon rank-sum test. Furthermore, we developed a regulon-associated specific score (RSS) tailored to a specific cell type by using the Jensen–Shannon divergence, which was computed with the assistance of the philentropy (v0.6.0) package.

### 2.13. Cell–Cell Communications

To explore potential interactions among diverse cell types within the ccRCC TME, we conducted a cell–cell communication analysis utilizing the CellPhoneDB Python package (v2.1.1) as previously described. By default, only those ligands and receptors that were expressed in at least 10% of cells within a specific cell type were included. Additionally, only statistically significant pairs with a *p* value < 0.05 were included in subsequent analyses.

To further pinpoint the crucial mediators of two cell subgroups, we used the NicheNet package to deduce the interaction between high-NS malignant cells and OLR1^+^ macrophages. The Top 20 ligands and Top 100 targets of differentially expressed genes of “sender cells” and “receiver cells” were extracted for paired ligand–receptor activity analysis. A NicheNet_output$ligand_activity_target_heatmap was generated to plot ligand regulatory activity. The expression levels of differentially expressed ligands and receptors were also depicted in a heat map, where the average level of a gene's expression in a specified cell type was scaled across the indicated subtypes.

### 2.14. Survival Analysis

A Kaplan–Meier (KM) analysis was performed to compare the overall survival (OS) rates and progression-free survival (PFS) times of the low- and high-NS groups by utilizing the survminer and survival R packages.

### 2.15. Spatial Transcriptomics Analysis

The spatial transcriptomics datasets were analyzed using the Seurat package (v4.1.1) in R. Normalization across spots was performed using the LogVMR function. Dimensionality reduction and clustering were carried out using PCA, with the first 30 PCs being considered. A signature score derived from the scRNA-seq dataset was added to the “metadata” of the ST dataset with the “AddModuleScore” function and using the default parameters in Seurat. Spatial feature expression plots were generated using the “SpatialFeaturePlot” function in the Seurat package.

### 2.16. Cell Culture

Human ccRCC 786-O cells and murine ccRCC Renca cells were purchased from Jinyuan Biotechnology Co., Ltd. (Shanghai, China). 786-O cells were maintained in RPMI-1640 medium (Gibco, United States) supplemented with 10% FBS (Gibco, United States) and 1% penicillin/streptomycin (Sigma, United States) at 37°C under 5% CO_₂_. Hypoxic exposure (1% O₂) was applied to 786-O cells for 12–24 h. Renca cells were cultured in DMEM (Gibco, United States) containing 10% FBS and 1% penicillin/streptomycin under identical conditions.

### 2.17. Cell Proliferation Assays

Three complementary assays were employed to evaluate cellular proliferation: the CCK-8 assay, 5-ethynyl-2⁣′-deoxyuridine (EdU) incorporation assay, and colony formation assay. All experiments were independently repeated four times (*n* = 4) unless otherwise specified. 786-O cells were seeded in 96-well plates (2 × 10^3^ cells/well) and cultured in RPMI-1640 medium containing 10% FBS. After 0/24/48/72/96 h incubation, 10 *μ*L CCK-8 reagent (Apexbio, United States) was added to each well. Absorbance at 450 nm was measured 2 h later using a microplate reader (BioTek, United States). Alternatively, for EdU incorporation, cells were pulse-labeled with 10 *μ*M EdU (Beyotime, China) for 2 h, fixed with 4% paraformaldehyde, and processed using the Cell-Light EdU Kit. Nuclei were counterstained with Hochest33342. EdU-positive cells were imaged by fluorescence microscopy (Nikon, Japan) and quantified across ≥ 3 random fields. For the colony formation assay, cells (500/well) were plated in 6-well plates and cultured for 10 days. Colonies were fixed with 4% paraformaldehyde, stained with 0.1% crystal violet (Sigma, United States), and quantified using ImageJ software. Clusters containing > 50 cells were counted as valid colonies.

### 2.18. Subcutaneous Renal Cancer Model Establishment in BALB/c Mice

Renca cells stably transfected with LV-Con or LV-Ets1 were cultured in RPMI-1640 medium supplemented with 10% FBS and 1% penicillin/streptomycin. Cells in the logarithmic growth phase were harvested, centrifuged (300 × g, 5 min), and resuspended in sterile PBS. Cell viability (> 95%) was confirmed by trypan blue exclusion, and suspensions were adjusted to 1 × 10^7^ cells/mL. Female BALB/c mice (6–8 weeks old) were randomly allocated into two groups (LV-Con vs. LV-Ets1, *n* = 6/group). Under aseptic conditions, 100 *μ*L cell suspension (1 × 10^6^ cells) was injected subcutaneously into the right flank using a 27-gauge needle. Tumor growth was monitored every 3 days by measuring perpendicular diameters (length (*L*) and width (*W*)) with calipers; volume was calculated as (L × W^2^)/2. At 28 days post-inoculation, mice were euthanized by intraperitoneal sodium pentobarbital (150 mg/kg) in accordance with institutional ethical guidelines. Carcasses were segregated, clearly labeled as barbiturate-containing, and disposed of via a licensed contractor by high-temperature incineration, in compliance with institutional biosafety procedures and local regulations; soiled bedding and disposable materials were managed as biohazardous waste and autoclaved before final disposal. Tumors were excised, weighed, and photographed.

### 2.19. Fluorescence-Activated Cell Sorting (FACS) Analysis

Single-cell suspensions from BALB/c mouse subcutaneous tumors were prepared via enzymatic digestion and filtered through a 70-*μ*m strainer. Each group consisted of five independent experiments (*n* = 5), with tumor tissue from a single mouse used for each independent experiment. Primary sorting was performed using fluorochrome-conjugated antibodies: BV421 anti-mouse CD11b (562605, BD Pharmingen) and FITC anti-mouse OLR (ab81710, Abcam). OLR^+^CD11b^+^ macrophages were gated and sorted on a BD FACSAria III cell sorter. For subset isolation, sorted OLR^+^CD11b^+^ macrophages were fixed and stained intracellularly with APC anti-mouse iNOS (17-5920-82, Thermo Fisher) or surface-stained with PE anti-mouse CD206 (141706, BioLegend). Proinflammatory (iNOS^+^) and anti-inflammatory (CD206^+^) subsets were subsequently sorted using stringent gating strategies.

### 2.20. Quantitative Real-Time Polymerase Chain Reaction (qRT-PCR)

Total RNA was extracted utilizing TRIzol reagent (Invitrogen, United States). Subsequently, the concentrations of RNA were assessed by optical density measurements. Reverse transcription of mRNA (Vazyme, China) was performed according to Vazyme's instructions. Real-time PCR amplification was carried out in triplicate. ChamQ SYBR qPCR Master Mix (Vazyme) was used to create cDNA fragments for use in quantitative PCR. *β*-Actin was used to normalize the levels of mRNAs. Relative mRNA expression was assessed using the 2-*ΔΔ*Ct method. Each experiment was independently repeated four times (*n* = 4).

### 2.21. Western Blot Analysis

Samples were lysed in RIPA buffer (Thermo, United States). The protein concentration was determined by BCA assay (Beyotime, China). Equal amounts of protein (20 *μ*g) were separated by SDS-PAGE and subsequently transferred to a PVDF membrane (Millipore, United States). The membrane was blocked for 1 h at RT and then incubated with primary antibodies overnight at 4°C. The following day, the membrane was incubated with enzyme-labeled secondary antibodies for 1 h at RT and exposed to ECL exposure solution (Thermo, United States). Finally, visualization of proteins was obtained using an Amersham Imager 600 system (GE Healthcare, United States). The immunostaining intensity of protein blots was measured using ImageJ software. All western blot experiments were independently repeated three times (*n* = 3).

### 2.22. Extracellular Acidification Rate (ECAR) Analysis

786-O cells were seeded at 8000 cells/well in XF96 microplates (Agilent, United States) and cultured overnight in RPMI-1640 medium supplemented with 10% FBS. Prior to analysis, cells were washed and equilibrated for 1 h in unbuffered XF assay medium (Agilent, 103334-100; pH 7.4) containing 2 mM L-glutamine. Glycolytic flux was assessed using a Seahorse XFe96 Analyzer (Agilent) with sequential injections of 10 mM glucose, 1 *μ*M oligomycin, and 50 mM 2-deoxy-D-glucose. Real-time ECAR (mpH/min) was recorded over three measurement cycles following each injection. All ECAR assays were independently performed four times (*n* = 4) to ensure reproducibility.

### 2.23. Statistical Analysis

All statistical analyses were performed as described in Seurat (v4.1.1) within the R (v4.2.0) software environment. Spearman's correlation was used to assess correlations. A survival analysis was conducted using the KM method. The Benjamini–Hochberg method was used to make multiple comparison corrections in the Wilcoxon's rank-sum test. Intergroup comparisons were conducted using unpaired two-tailed Student's *t*-tests for two-group analyses, whereas one-way ANOVA was implemented for multigroup comparisons. Statistical significance was set at *p* < 0.05.

## 3. Results

### 3.1. Identification of NS-Related Molecular Subtypes in ccRCC

**Figure 1:**
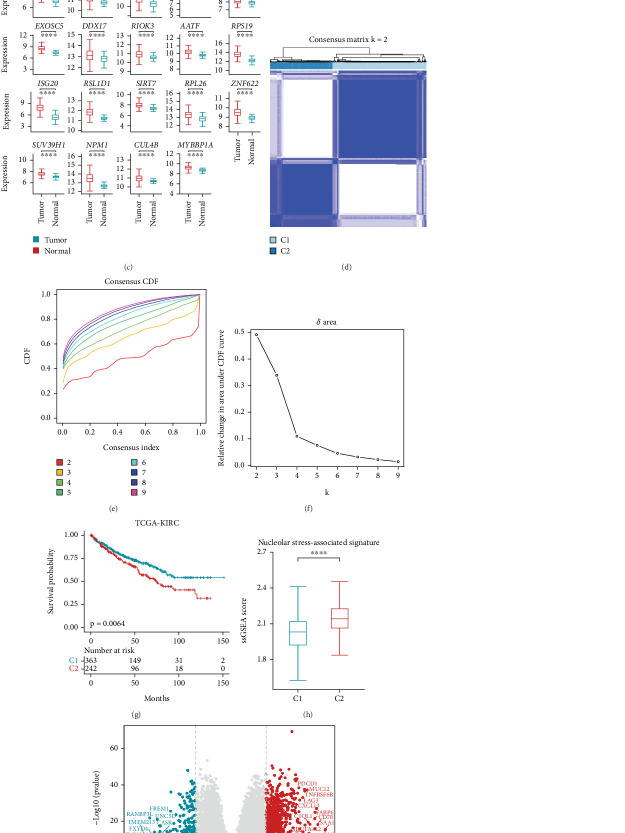
Identification of NS-related molecular subtypes in ccRCC. (a) Venn plot showing the 38 NS-associated genes. (b) Heatmap showing the expression of the identified 38 NS-associated genes in samples annotated by sample types. Color scale: blue denoted low expression, and red denoted high expression. (c) Box plots showing the expressions of 19 significantly upregulated genes (*LYAR, EIF2A, RPS27L, REXO4, RRP8, EXOSC5, DDX17, RIOK3, AATF, RPS19, ISG20, RSL1D1, SIRT7, RPL26, ZNF622, SUV39H1, NPM1, CUL4B,* and *MYBBP1A*). Different colors represent different sample types. (d) Consensus clustering based on 19 significantly upregulated NS-associated genes. Light blue: C1 with low NS score, dark blue: C2 with high NS score. (e) Empirical CDFs correspond to the entries of consensus matrices K from 2 to 9. (f) Proportion increases the delta in the area under the CDF. (g) Survival analysis of the patients grouped by C1 and C2 in the TCGA-ccRCC cohort. (h) Box plots showed the NG-associated signature ssGSEA scores in C1 and C2 samples in the TCGA-ccRCC dataset. (i) Differential expressing genes analysis between C1 and C2. (j) Radar plots showing the Top 10 KEGG and GO BP terms enriched in the C1 (blue) and C2 (red). A two-sided Wilcoxon test computed *p* values. ⁣^∗^*p* ≤ 0.05, ⁣^∗∗^*p* ≤ 0.01, ⁣^∗∗∗^*p* ≤ 0.001, and ⁣^∗∗∗∗^ *p* ≤ 0.0001.

Disruption of ribosome biogenesis leads to NS, which subsequently activates molecular systems aimed at restoring cellular homeostasis [17–19]. Perturbations at any stage of nucleolar ribosome biogenesis can induce NS, a condition closely associated with cancer [17, 20, 21]. Cancer cells are particularly susceptible to NS, which can enhance ribosome biosynthesis and support their growth and proliferation [20, 22, 23]. To evaluate the role of NS in ccRCC, we identified 38 genes by intersecting ribosome biogenesis and cellular stress response pathways, defining this set as the NS-associated signature (Figure 1a). A heat map illustrated differential expression of the NS-associated signature between tumor and normal samples in the TCGA-ccRCC cohort (Figure 1b). After multiple iterations of differential expression analysis, 19 stable and tumor-specific upregulated NS-associated genes were identified (Figure 1c and Figure S1a). Consensus clustering revealed two distinct NS-associated gene expression modules in the TCGA-ccRCC cohort (Figures 1d, 1e, and 1f). Survival analysis demonstrated that patients in Cluster 1 (C1) had a more favorable prognosis than those in Cluster 2 (C2) (Figure 1g). Additionally, C2 exhibited a high ssGSEA score for the NS-associated signature, classifying it as the high-NS subtype, whereas C1 showed a low score and was defined as the low-NS subtype (Figure 1h).

Next, we performed differential expression and functional enrichment analyses to further investigate the molecular mechanisms underlying NS in ccRCC. The high-NS subtype exhibited an immunosuppressive phenotype, characterized by elevated expression of immune checkpoint molecules (*PDCD1*, *LAG3*, and *CD70*), chemokines (*CXCL13*), tumor markers (*CA9*), and fatty acid metabolism–related genes (*FABP6* and *FABP7*) (Figure 1i). Functional enrichment analysis revealed that upregulated genes in the high-NS subtype were primarily associated with immune-related processes (Figure 1j and Figure S1b). In contrast, the low-NS subtype was enriched in pathways related to metabolism and transport, including bile secretion, tight junctions, and inorganic anion transport (Figure 1j). These findings suggest that elevated NS is associated with an immunosuppressive TME.

### 3.2. Genomic Landscape in the Low- and High-NS Subtypes

**Figure 2:**
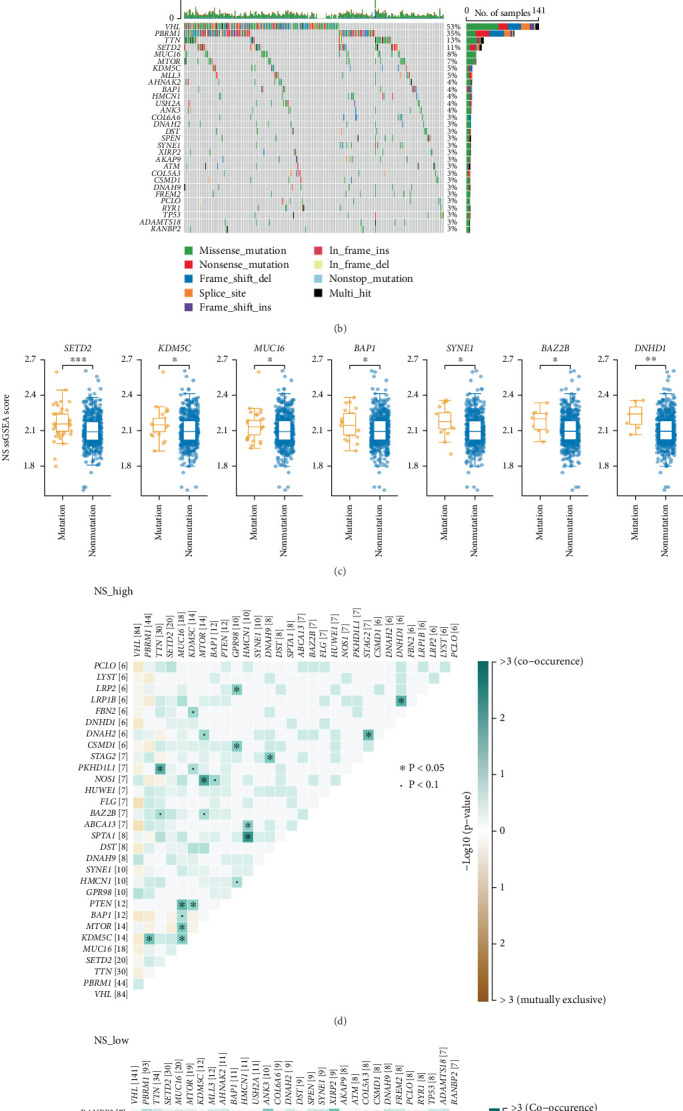
The genomic landscape between the low- and high-NS subtypes. (a, b) Comparison of the variation in the mutation status of the Top 30 genes with mutations between (a) high-NS and (b) low-NS. (c) Box plots show that *SETD2*, *KDM5C*, *MUC16*, *BAP1*, *SYNE1*, *BAZ2B*, and *DNHD1* gene mutations were considerably linked to the high-NS signature (Wilcoxon rank-sum test, ⁣^∗^*p* ≤ 0.05, ⁣^∗∗^*p* ≤ 0.01, and ⁣^∗∗∗^*p* ≤ 0.001). (d, e) Concurrence (blue) and mutual exclusion (brown) between high-frequency mutation genes (the Top 30 genes with mutations) in (d) high-NS and (e) low-NS groups; the number following the gene names represents the number of samples with this specific gene mutation. TMB, tumor mutational burden.

To investigate the genomic landscape associated with NS subtypes, we compared tumor-intrinsic mutations within the TCGA-ccRCC cohort. The high-NS group exhibited significantly higher mutation frequencies in *PTEN*, *GPR98*, *BAZ2B*, and *DNHD1* compared to the low-NS group (Figure 2a,b). Moreover, the ssGSEA score for the NS-associated signature was markedly elevated in samples harboring mutations in *SETD2*, *KDM5C*, *MUC16*, *BAP1*, *SYNE1*, *BAZ2B*, and *DNHD1* relative to their wild-type counterparts (Figure 2c).

We further explored patterns of co-occurrence and mutual exclusivity among frequently mutated genes. In the high-NS subtype, *SPTA1* mutations co-occurred with *HMCN1* mutations, while *PTEN* mutations frequently co-occurred with either *MUC16* or *KDM5C* mutations (Figure 2d). In contrast, in the low-NS subtype, *TP53* mutations co-occurred with *AHNAK2* or *SPEN* mutations, and *DNAH9* mutations co-occurred with *AHNAK2*, *MTOR*, or *SPEN* mutations (Figure 2e).

### 3.3. Immune Infiltration Characteristics

**Figure 3:**
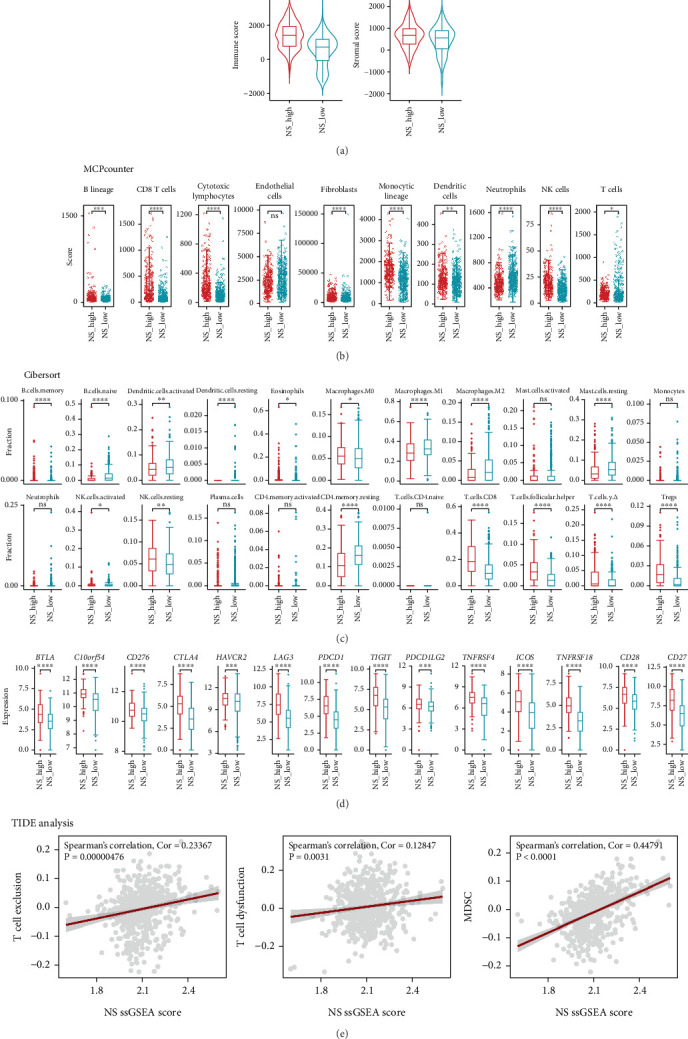
Immune infiltration landscape in high-NS and low-NS subtypes. (a) Box plots showing the (A) immune and (B) stromal scores of the high (red) and low (blue) NS subtypes. (b) Box plots showing the 10 cell types' infiltration identified by the MCPcounter method in the high (red) and low (blue) NS subtypes. Wilcoxon rank-sum test was used to assess the difference. (c) Immune infiltration of 22 immune cell types of the high (red) and low (blue) NS subtypes by the cibersortx method. (d) Box plot showing the 14 immune checkpoint-associated genes between the high (red) and low (blue) NS subtypes. (e) Correlation analysis between the NS-associated signature and the T cell exclusion, T cell dysfunction, and MDSC signature inferred by the TIDE method. Correlation analysis was calculated using Spearman's rank correlation coefficient.

Accumulating evidence suggests that NS plays a pivotal role in modulating the immune system [24]. To examine this, we analyzed the composition of the TME. Overall, our data showed that the immune score was significantly higher in the high-NS group compared to the low-NS group (Figure 3a). Furthermore, patients in the high-NS group exhibited increased proportions of B lineage cells, CD8^+^ T cells, cytotoxic lymphocytes, monocytic lineage cells, and natural killer (NK) cells. In contrast, the low-NS group showed higher infiltration of T cells, neutrophils, and endothelial cells (Figure 3b). Subsequent CIBERSORTx analysis of 22 immune cell types in the TME revealed that the high-NS subtype exhibited elevated levels of resting regulatory T cells, CD8^+^ T cells, follicular helper T cells, and NK cells, along with reduced levels of M2 macrophages, resting CD4^+^ memory T cells, and activated dendritic cells (Figure 3c). Additionally, numerous immune checkpoint–related genes were upregulated in the high-NS subtype (Figure 3d). Significant positive correlations were observed between the NS-associated signature and T cell exclusion, T cell dysfunction, and MDSC signatures in ccRCC (Figure 3e; Spearman's correlation test: *ρ* = 0.23367, *p* = 0.00000476; *ρ* = 0.12847, *p* = 0.0031; and *ρ* = 0.44791, *p* < 0.0001), suggesting that patients in the high-NS subtype may experience diminished benefit from immunotherapy.

### 3.4. The Association Between NS and Cells at the Single-Cell Level

**Figure 4:**
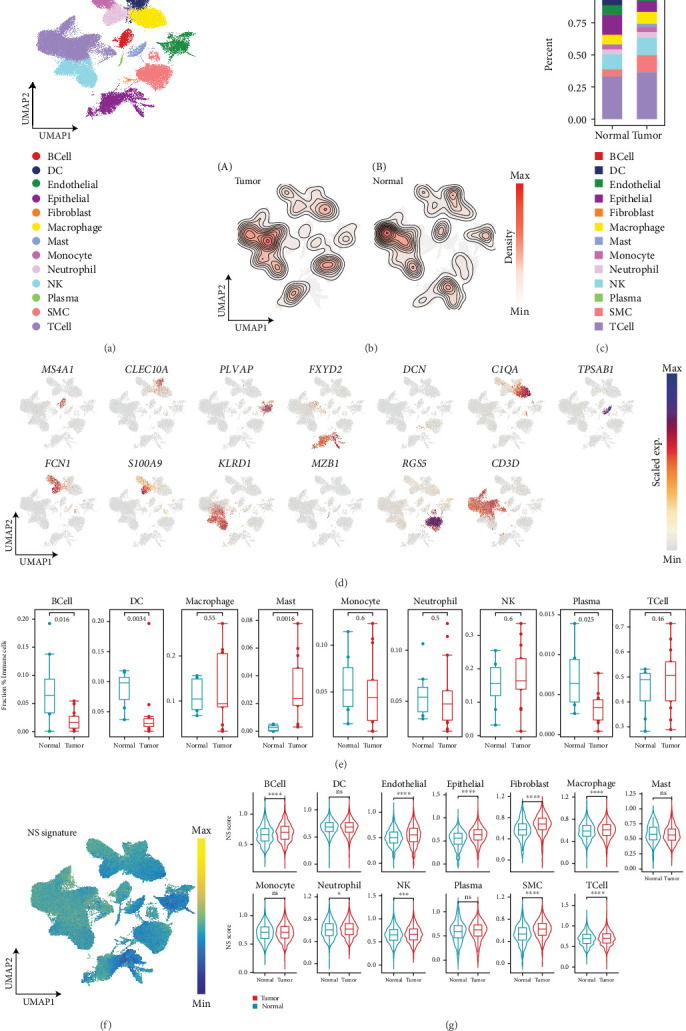
Single-cell RNA-seq analysis of ccRCC samples. (a) UMAP plot showing the classification of cell types, with different colors representing different cell types. DC, dendric cells; NK, natural killer cells; SMC, smooth muscle cells. (b) 2D density plots showing major cell population enrichment in tumor (A) and normal (B) samples. (c) The proportion of the cell types in differential sample types is shown in bar plots, with different colors representing different cell types. (d) UMAP plots show the expression of classical molecular markers of corresponding cell types; the color depth represents the marker expression value. (e) Box plots showing the ratio of the major cell types in all immune cells between tumor (red) and normal (blue) samples. (f) UMAP plots show the expression of the NS-associated signature; the color depth represents the signature value. (g) Box plots showing the expression of the NS-associated signature between tumor (red) and normal (blue) samples in the major cell types.

To comprehensively investigate the cellular composition of the ccRCC microenvironment, we reanalyzed the scRNA-seq dataset GSE178481, which includes eight normal and 13 tumor samples, as published by Alchahin et al. [25]. The Harmony algorithm was used to correct for batch effects (Figure S2a,b). After quality control, 128,179 cell transcriptomes were retained for downstream analysis and clustered into 21 distinct groups representing 13 different cell subtypes (Figure 4a and Figure S2a). These subtypes were identified using canonical single-cell markers, including B cells (*MS4A1* and *CD79A*), dendritic cells (*FCER1A*, and *CLEC10A*), endothelial cells (*CLDN5* and *VMF*), epithelial cells (*KRT8* and *KRT19*), fibroblasts (*DCN* and *COL1A2*), macrophages (*C1QB* and *C1QA*), mast cells (*CPA3* and -*KIT*), monocytes (*FCN1* and *CD14*), neutrophils (*S100A9* and *S100A8*), NK cells (*GNLY* and *GZMB*), plasma cells (*MZB1* and *IGKC*), smooth muscle cells (*ACTA2* and *TAGLN*), and T cells (*CD3D* and *CD3E*) (Figure 4d and Figure S2c). A comparison of cell subtype infiltration across sample types revealed significant differences in immune cell composition. Tumor samples were enriched in immune cells—particularly macrophages, mast cells, and neutrophils—but contained fewer stromal cells, such as smooth muscle and endothelial cells, compared to normal samples (Figure 4b,c). We then computed fractional contributions to identify additional cell types altered in the immune microenvironment and performed subsequent differential analysis (Figure 4e). Notably, mast cell numbers were increased, whereas B cells, dendritic cells, and plasma cells were decreased in tumor samples.

To evaluate NS across cell types, we mapped the expression patterns of the NS-associated signature across various cell populations (Figure 4f and Figure S2d). Our results revealed significantly elevated NS-associated signature expression in immune cell populations. Furthermore, NS-associated signature scores were higher in tumor tissues compared to adjacent normal tissues (Figure 4g). These findings are consistent with previous studies and underscore the critical role of NS in ccRCC progression.

### 3.5. Transcriptome Analysis of Malignant Cells Based on the NS-Associated Signature

**Figure 5:**
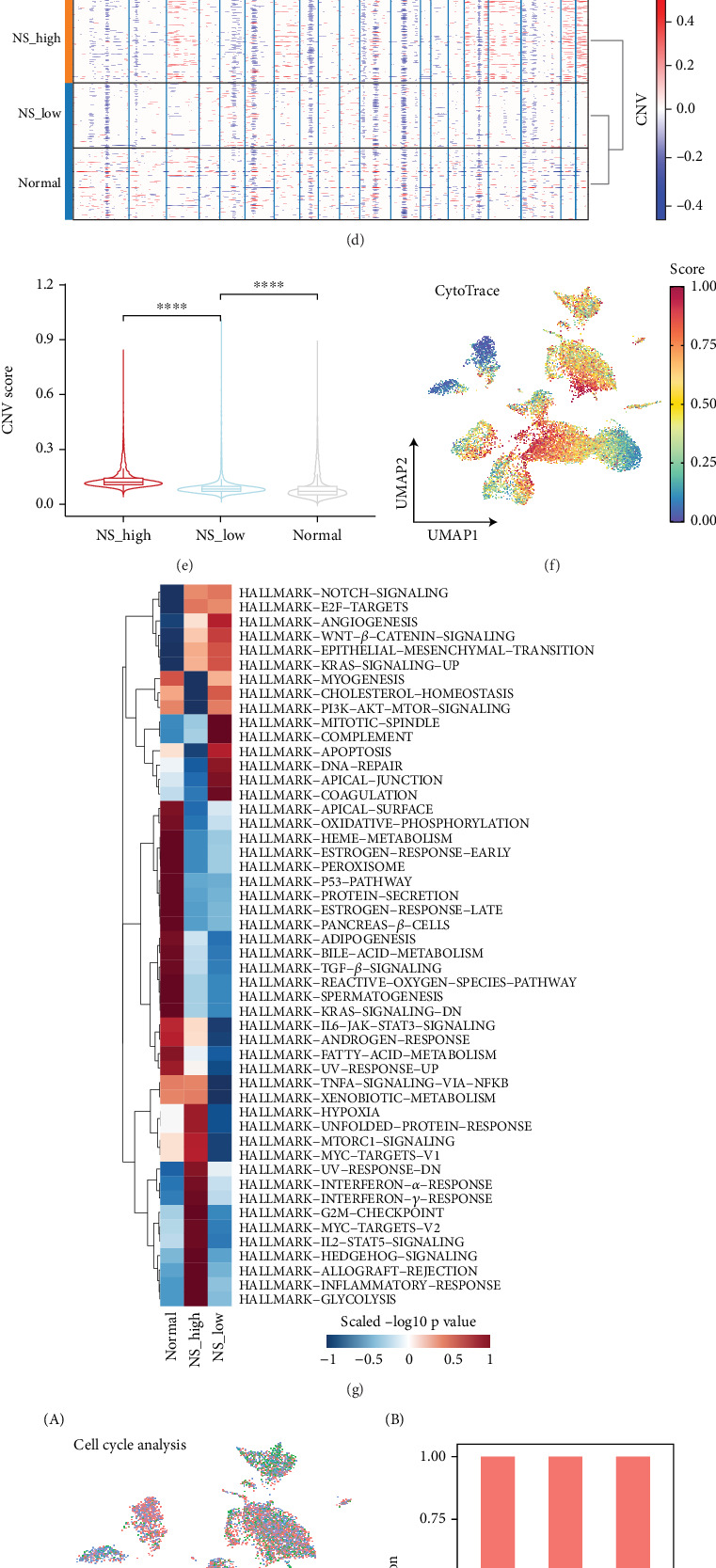
Transcriptome analysis of malignant cells based on the NS-associated signature. (a) UMAP plot showing the classification of malignant subtypes based on the NS-associated signature, with different colors representing different subtypes. (b) UMAP plot showing the distribution of sample types, with different colors representing different sample types. (c) The UMAP plot shows the NS-associated signature distribution; (A) the color depth represents the signature value, and (B) the violin plot shows the NS-associated signature in the malignant subtypes. (d) Representative CNV heat map with hierarchical clustering of results from inferCNV analysis in the malignant subtypes. (e) The violin plots and box plots show the CNV score in the malignant subtypes, with different colors representing different malignant subtypes. (f) The UMAP plot shows the CytoTrace-inferred score distribution; the color depth represents the signature value. (g) Heatmap showing the average activity of hallmark pathways in the malignant subtypes. (h) UMAP plot showing the distribution of G1, G2/M, and S phases, (A) with different colors representing cell cycle phases, and the proportion of the cell cycle phases in the malignant subtypes is shown in bar plots, (B) with different colors representing different cell cycle phases. (i) Differential expressing genes analysis between high- and low-NS subgroups. (j) Heatmap showing the enriched KEGG pathways in the malignant subtypes.

To further explore the relationship between malignant cells and the NS-associated signature, we reclustered epithelial cells into 15 distinct clusters (Figure S3a). Based on the distribution of sample types and NS-associated signature scores, we categorized epithelial cells as normal, high-NS malignant, or low-NS malignant (Figures 5a, 5b, and 5c and Figure S3b,c). Notably, high-NS malignant cells exhibited significant chromosomal amplifications on Chromosomes 3, 7, 17, 20, and 21, along with deletions on Chromosomes 6 and 12 (Figure 5d). Furthermore, the high-NS malignant group demonstrated more pronounced copy number variation features than the low-NS malignant and normal epithelial groups (Figure 5e).

We applied the CytoTRACE method to infer the developmental plasticity of malignant cells, which indicated that high-NS malignant cells may exhibit increased stemness features and more aggressive malignant potential. Differential expression analysis of the three epithelial subtypes revealed that genes upregulated in the high-NS malignant group were enriched in hallmark pathways associated with cell proliferation. Cell cycle analysis indicated that a greater proportion of high-NS malignant cells were in the G2/M phase, further supporting their proliferative phenotype (Figure 5h).

Differential expression and functional enrichment analyses identified elevated expression of ribosome-associated genes (*RPS26* and *RPL21*), chemokines (*CCL2* and *CCL4*), and metabolism-related genes (*NNMT*, *PDK4*, and *NDUFA4L2*) in high-NS malignant cells (Figure 5i). Genes upregulated in high-NS malignant cells were also enriched in pathways including ribosome biogenesis, TNF signaling, and glycolysis/gluconeogenesis (Figure 5j). These results suggest that high-NS malignant cells possess enhanced proliferative capacity and are likely involved in glycolytic metabolism.

### 3.6. The Trajectory of Cell–State Transitions Among NS-Associated Malignant Subpopulations

**Figure 6:**
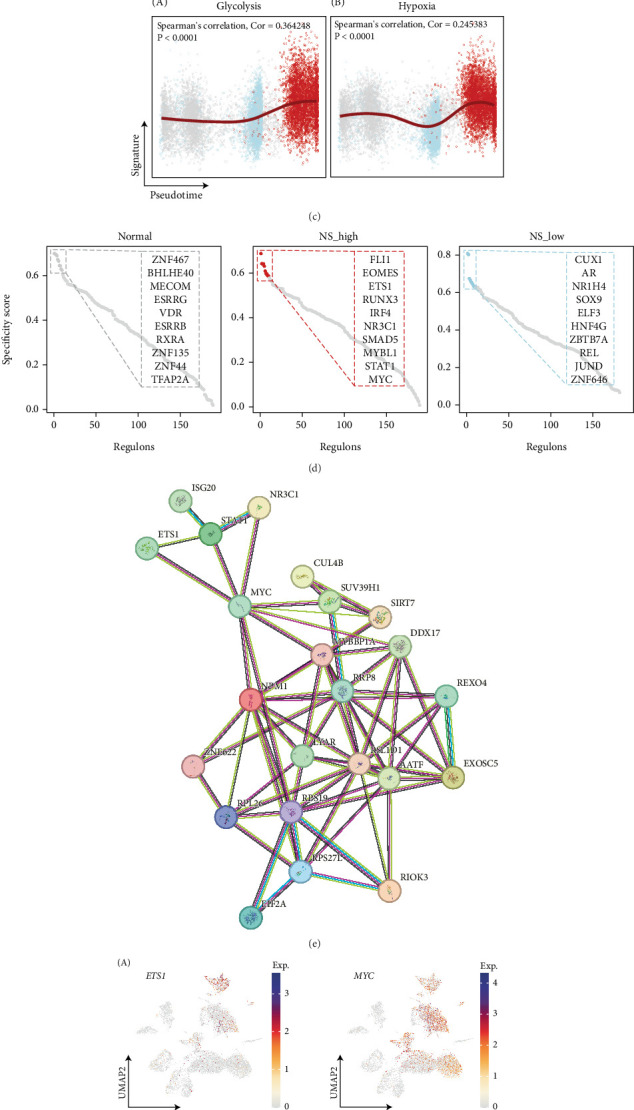
Trajectory analysis of malignant subtypes in ccRCC. (a) The developmental trajectory is inferred by the scTour and colored according to the subtypes. (b) The pseudotime of the developmental trajectory. Colors from purple to yellow indicate the value from low to high. (c) Dot plot showing Spearman's correlation of the pseudotime and glycolysis (A) and hypoxia (B) score. (d) Dot plots showing the Top 10 specific activated TFs ranked by the regulon-specific score (RSS) in each malignant subtype. (e) Protein–protein interaction (PPI) network based on ETS1, MYC, and 19 tumor-specific upregulated NS-associated genes. (f) UMAP plots (A) showing the expression of *ETS1* and *MYC*. UMAP plots (B) showing the activity of *ETS1* and *MYC* regulon.

To gain insights into the dynamic mechanisms underlying malignant progression at the single-cell level, we applied the scTour algorithm to derive a pseudotime cell trajectory (Figure 6a). Integration of pseudotime and inferred cell–state statistics revealed distinct transition pathways, with normal epithelial cells occupying the progenitor state and diverging into high-NS malignant cells (Figure 6a,b). We further examined glycolysis and hypoxia pathway signatures along pseudotime in the context of tumor evolution (Figure 6c; Spearman's correlation: *ρ* = 0.364, *p* < 0.0001 and *ρ* = 0.245, *p* < 0.0001). Given the central role of hypoxia in ccRCC, we conducted a pySCENIC analysis to identify key TFs and their regulatory activities. We then computed RSSs for the high-NS malignant group using Jensen–Shannon divergence and identified ETS1 and MYC among the Top 10 TFs ranked by RSSs (Figures 6d, 6e, and 6f). Previous research by Oikawa et al. [26] demonstrated that ETS1 plays a crucial role in angiogenesis and cancer invasion and is transcriptionally regulated by hypoxia via HIF-1*α*. These findings suggest that activation of ETS1 and MYC may drive malignant progression under hypoxic conditions. Furthermore, we constructed a protein–protein interaction network based on ETS1, MYC, and 19 tumor-specific upregulated NS-associated genes (Figure 6e). The results indicate that ETS1 activation may induce NS during hypoxia through the ETS1–MYC–NPM1/DDX17/SIRT7 axis.

### 3.7. ETS1 Drives Hypoxia-Induced ccRCC Progression via the MYC/NPM1/DDX17 Axis

**Figure 7:**
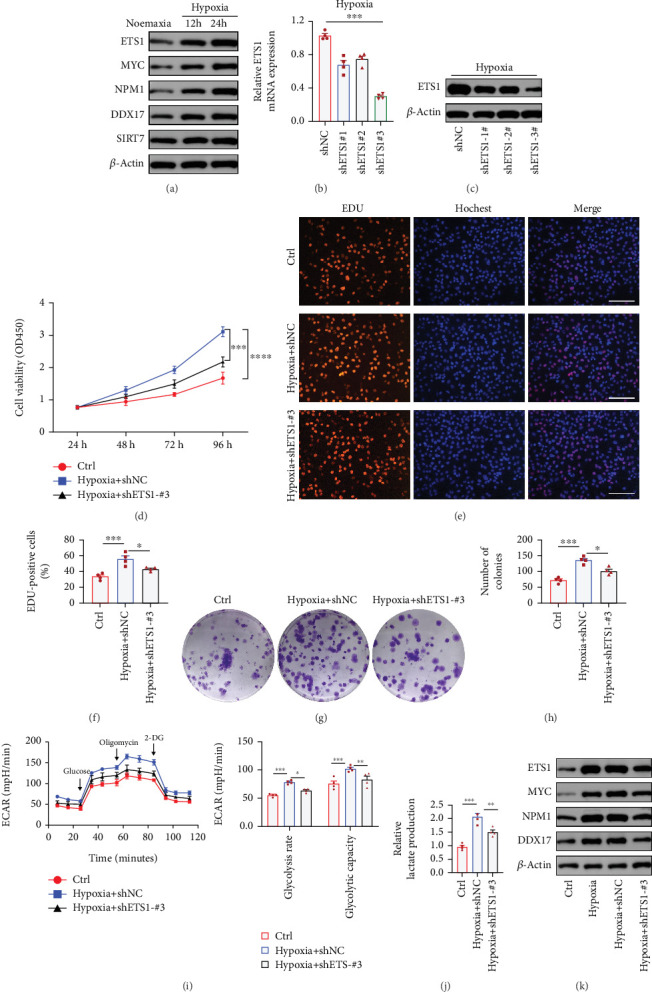
ETS1 drives hypoxia-induced ccRCC progression via the MYC/NPM1/DDX17 axis. (a) Western blot analysis of ETS1, MYC, NPM1, DDX17, and SIRT7 expressions in 786-O cells under hypoxic conditions (1% O_₂_) for 12-24 h. (b, c) Quantitative analysis of ETS1 mRNA and protein levels in 786-O cells following ETS1 knockdown under hypoxic conditions. Cells were transfected with a nontargeting control shRNA (shNC) or three distinct shRNA constructs targeting ETS1 (shETS1-#1 to shETS1-#3). *n* = 4 per group. (d) CCK-8 assay assessing proliferative activity in three experimental groups: normoxic control (Ctrl), hypoxia with control shRNA (Hypoxia+shNC), and hypoxia with ETS1-targeting shRNA (Hypoxia+shETS1-#3) n = 4 per group. (e) EdU incorporation assay (red: EdU-positive cells; blue: Hoechst-stained nuclei) showing proliferation in Ctrl, Hypoxia+shNC, and Hypoxia+shETS1-#3 groups. Scale bar: 100 *μ*m. (f) Statistical analysis of EdU-positive cells from (e). n = 4 per group. (g) Colony formation assay evaluating proliferation in the three experimental groups. (h) Quantification of colony numbers from (g). n = 4 per group. (i) Glycolytic rate (ECAR) measured by Seahorse XF Analyzer in the three groups. n = 4 per group. (j) Lactate production determined using a lactate assay kit. n = 4 per group. (k) Western blot analysis of ETS1, MYC, NPM1, and DDX17 protein levels in four groups: Ctrl, Hypoxia, Hypoxia+shNC, and Hypoxia+shETS1-#3. Note: Hypoxia exposure was maintained at 1% O_₂_ throughout experiments. Data were presented as mean ± SEM. ⁣^∗^*p* < 0.05, ⁣^∗∗^*p* < 0.01, ⁣^∗∗∗^*p* < 0.001, and ⁣^∗∗∗∗^*p* < 0.0001.

Building on our preceding single-cell and spatial transcriptomic analyses, we identified ETS1 as one of the top TFs specifically activated in high-NS malignant cells. ETS1 activity was strongly associated with NS via the MYC/NPM1/DDX17/SIRT7 signaling axis, and these high-NS malignant cells displayed pronounced proliferative capacity, glycolytic metabolic reprogramming, and potential for immunosuppressive niche formation. To experimentally validate the functional role of ETS1 in this context, human ccRCC 786-O cells were cultured under hypoxic conditions (1% O_2_) for 12–24 h. We observed a time-dependent increase in ETS1 expression, accompanied by activation of the MYC/NPM1/DDX17 axis under hypoxia, while SIRT7 expression remained unchanged (Figure 7a). To investigate the regulatory role of ETS1 in hypoxia-induced ccRCC proliferation, we performed *ETS1* knockdown in 786-O cells (Figure 7b,c). As shown in Figure 7d, hypoxia significantly enhanced proliferative activity, which was markedly attenuated by *ETS1* silencing. Furthermore, *ETS1* depletion substantially reduced the proportion of EdU-positive cells (Figure 7e,f) and colony-forming capacity (Figure 7g,h). Hypoxia also promoted glycolytic metabolism in 786-O cells, whereas ETS1 inhibition significantly decreased both glycolytic rate and activity (Figure 7i), along with a reduction in lactate production (Figure 7j). Western blot analysis confirmed that ETS1 knockdown suppressed activation of the MYC/NPM1/DDX17 axis, potentially disrupting the immune-suppressive microenvironment mediated by high-NS malignant cells (Figure 7k).

### 3.8. Cell–Cell Interaction Between High-NS Malignant Cells and OLR1^+^ Macrophages in the Immunosuppressive TME

**Figure 8:**
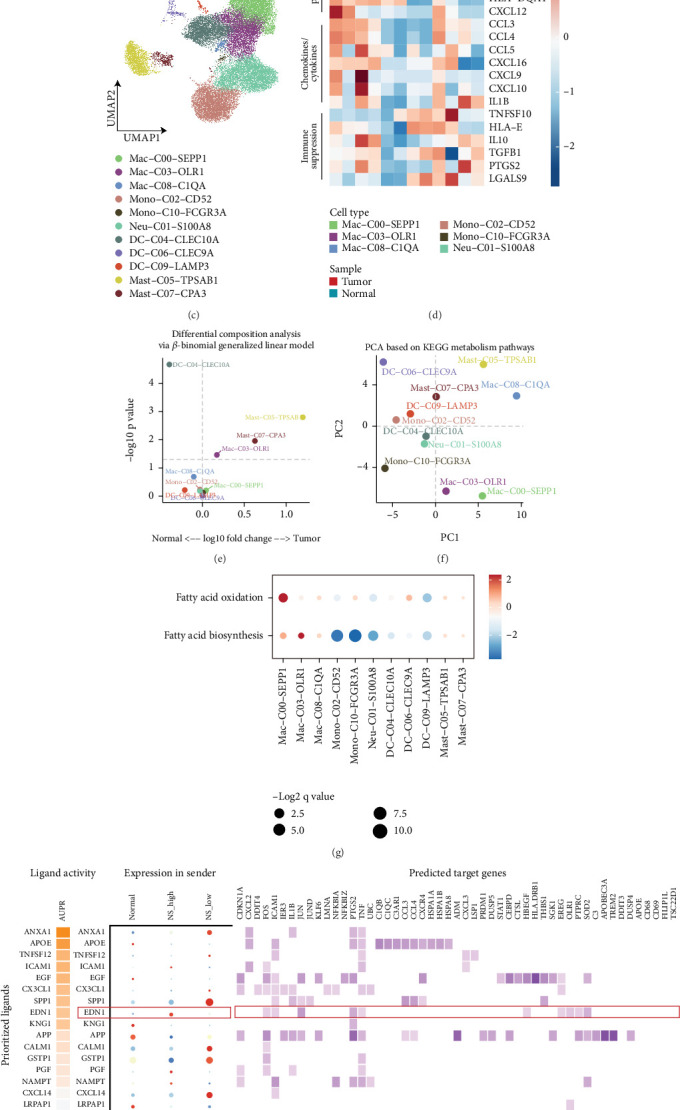
Single-cell analysis of myeloid cells in ccRCC. (a) Chord diagrams show the cell–cell interactions between major cell types in ccRCC. (b) Dot plot showing major cell types' outgoing and incoming interaction strength in ccRCC. (c) UMAP plot of individual myeloid cells. Each dot denotes one cell; color represents myeloid subtypes. (d) Comparison of the average expression of signature genes between cell subpopulations in tumor and normal groups. The top bar colors represent different cell subpopulations and sample types (tumor: red and normal: blue). Colors from blue to red represent the average expression from low to high. (e) Differential composition analysis via a beta-binomial generalized linear model between tumor and normal samples. (f) PCA of the myeloid cell subpopulations based on the scores of KEGG metabolism pathways. Dots and colors represent different cell subpopulations. (g) Comparison of the pathway enrichment of fatty acid oxidation and the biosynthesis process in the myeloid subpopulations. Statistics represent the enrichment degree; dot sizes represent the log-transformed *q*-value. FAO, fatty acid oxidation; FABP, fatty acid biosynthesis process. (h) Top-ranked ligands and ligand–target pairs are inferred to regulate the myeloid subpopulations by high-NS malignancy, according to NicheNet. (i) Density plots showing the myeloid subsets' proinflammatory scores (A) and immunosuppression scores (B). The central mark indicates the median, and the bottom and top edges of the box indicate the first and third quartiles, respectively.

To investigate how high-NS malignant cells regulate the immune microenvironment in ccRCC, we conducted a cell–cell interaction analysis using the CellPhoneDB method to evaluate communication between major cell types. We observed stronger interactions between high-NS malignant cells and myeloid cells compared to those among other cell populations (Figure 8a), suggesting that high-NS malignancy may contribute to an immunosuppressive microenvironment by modulating myeloid cell function. Specifically, high-NS malignant cells exhibited strong outgoing interactions, while myeloid cells—including dendritic cells (DCs), neutrophils, macrophages, and monocytes—displayed strong incoming interactions (Figure 8b). We further identified 11 myeloid subtypes based on marker gene expression: three macrophage subsets (Mac-C00-SEPP1, Mac-C03-OLR1, and Mac-C08-C1QA), two monocyte subsets (Mono-C02-CD52 and Mono-C10-FCGR3A), one neutrophil subset (Neu-C01-S100A8), three dendritic cell subsets (DC-C04-CLEC10A, DC-C06-CLEC9A, and DC-C09-LAMP3), and two mast cell subsets (Mast-C05-TPSAB1 and Mast-C07-CPA3) (Figure 8c and Figures S4a, S4b, S4c, and S4d). In addition to differences in cell abundance, scRNA-seq analysis revealed distinct transcriptional features in tumor samples compared to normal samples. Mac-C00-SEPP1 cells showed higher expression of phagocytosis-related markers (*MERTK*, *MRC1*, and *MSR1*) relative to Mac-C03-OLR1 and Mono-C10-FCGR3A cells, whereas Mac-C03-OLR1 cells expressed higher levels of immunosuppressive markers (*IL10*, *TGFB1*, and *PTGS2*) (Figure 8d). Notably, Mac-C03-OLR1 cells were significantly enriched in tumor samples, as determined by a beta-binomial generalized linear model (Figure 8e).

To further investigate the unique metabolic profiles of each macrophage subtype, we evaluated the activity of KEGG metabolic pathways. Our results revealed that Mac-C00-SEPP1, Mac-C03-OLR1, and Mac-C08-C1QA cells exhibited higher overall metabolic activity (Figure S4e). PCA showed that Mac-C00-SEPP1 and Mac-C03-OLR1 cells had metabolically distinct profiles compared to other subtypes (Figure 8f). Lipid droplet–dependent fatty acid metabolism has been shown to regulate the immunosuppressive phenotype of tumor-associated macrophages (TAMs); therefore, we examined the activity of fatty acid biosynthesis and oxidation pathways. The results indicated that Mac-C03-OLR1 cells displayed elevated fatty acid biosynthesis and reduced fatty acid oxidation activity relative to other subtypes (Figure 8g). Additionally, OLR1 (oxidized low-density lipoprotein receptor 1), a marker known for its role in lipid metabolism and tumor progression, was highly expressed in Mac-C03-OLR1 cells.

We applied the NicheNet framework to identify critical regulators mediating the interaction between high-NS malignant cells and Mac-C03-OLR1 cells in ccRCC. High-NS malignant cells exhibited strong ligand activity for EDN1 and high *EDN1* gene expression levels (Figure 8h). The EDN1 ligand was predicted to bind the EDNRA receptor and act on OLR1, which is expressed in Mac-C03-OLR1 cells (Figure 8g, Figure S4f, and Figure S5a). These findings suggest that high-NS malignancy promotes fatty acid biosynthesis in Mac-C03-OLR1 cells via the EDN1–EDNRA–OLR1 axis. Moreover, analyses of proinflammatory and immunosuppressive gene signatures revealed that Mac-C03-OLR1 cells exhibited enhanced immunosuppressive potential (Figure 8i). Altogether, these data support the existence of a functional interaction network between high-NS malignant cells and Mac-C03-OLR1 macrophages that may play a pivotal role in shaping the immunosuppressive TME in ccRCC.

### 3.9. ETS1-Mediated High-NS Tumor–Macrophage Interaction Drives Immunosuppression and Poor Prognosis in ccRCC

**Figure 9:**
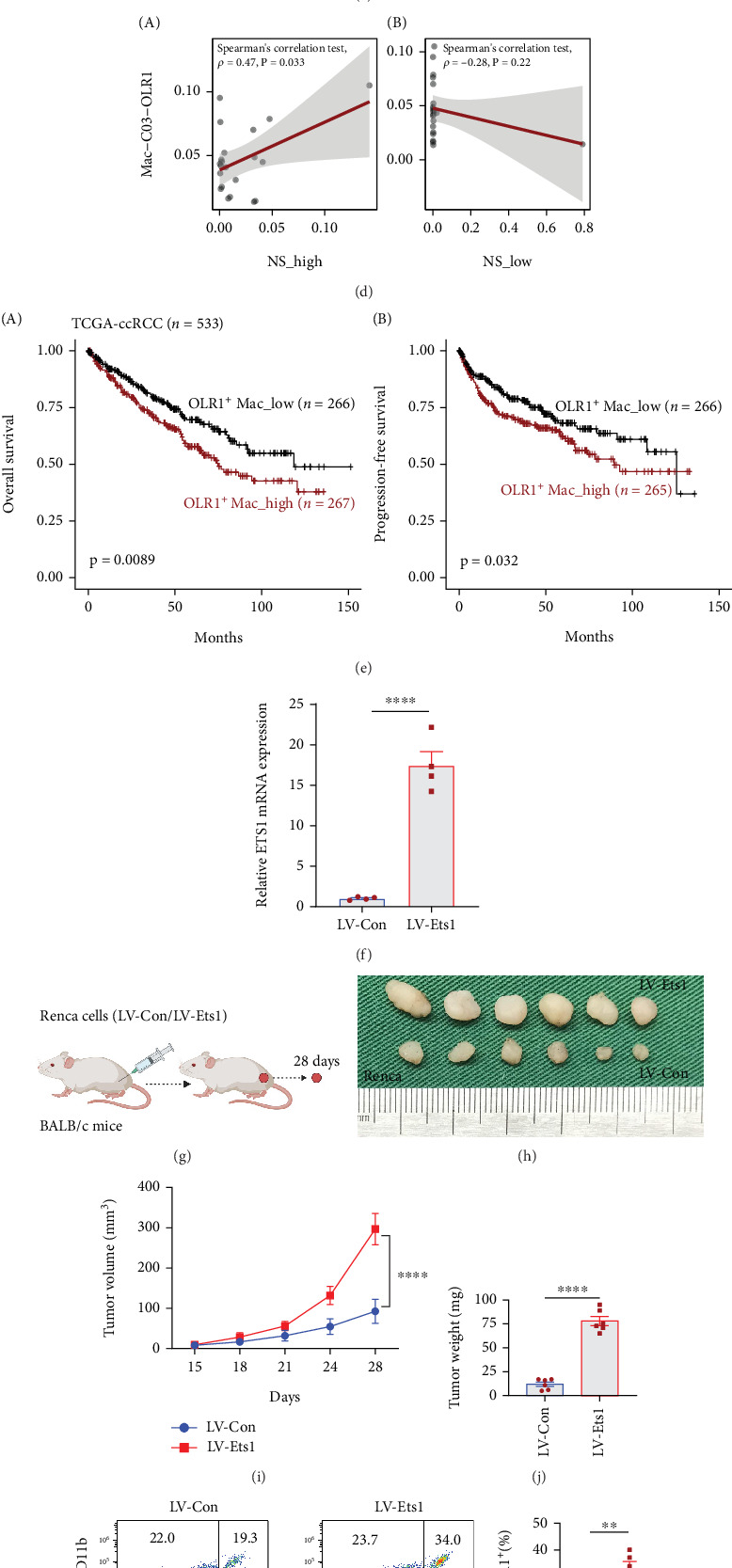
ETS1-mediated high-NS tumor malignant cell and OLR1+ macrophage interaction drives immunosuppression and poor prognosis in ccRCC. (a) Spatial feature plots of NS_low, NS_high, and OLR1^+^ Mac signature score in the ccRCC tissue (from left to right). (b) Spatial colocation of the EDN1-EDNRA pair. (c) Spatial feature plots showed the expression of CA9, OLR1, EDN1, and EDNRA in the ccRCC tissue (from left to right), respectively. (d) Dot plot showing Spearman's correlation of the OLR1^+^ Mac and (A) NS_high and (B) NS_low (score. (e) Overall (A) and progression-free (B) survival analyses for low and high infiltration of OLR1^+^ Mac patient groups in the TCGA-ccRCC cohort using Kaplan–Meier curves. (f) Generation of stable *Ets1*-overexpressing Renca cells using lentiviral transduction. (g) Schematic of the syngeneic BALB/c mouse subcutaneous renal cancer model established with Renca LV-Con and Renca LV-Ets1 cells. (h) Representative tumor images from the two groups in (G) (*n* = 6 mice/group). (i) Tumor growth curves (volume changes) in the two groups from (G) (*n* = 6 mice/group). (j) Tumor weight comparison between the two groups (*n* = 6 mice/group). (k) Infiltration density of OLR1^+^ macrophages in tumor tissues (*n* = 5 mice/group). (l) Quantification of OLR1^+^iNOS^+^ macrophages (M1-like phenotype) in tumor tissues (*n* = 5 mice/group). (m) Quantification of OLR1^+^CD206^+^ macrophages (M2-like phenotype) in tumor tissues (*n* = 5 mice/group). Note: LV-Con, lentiviral control; LV-Ets1, *Ets1*-overexpressing lentiviral construct. Macrophage subsets were analyzed via immunohistochemistry or flow cytometry using specific markers. Data were presented by mean ± SEM. ⁣^∗∗^*p* < 0.01 and ⁣^∗∗∗∗^*p* < 0.0001.

To investigate the spatial colocalization of high-NS malignant cells and OLR1^+^ macrophages in ccRCC, we applied the AddModuleScore algorithm to map the distribution of high-NS malignant cells, low-NS malignant cells, and OLR1^+^ macrophages in spatial transcriptomic samples. We observed substantial tumor heterogeneity in the spatial arrangement of low- and high-NS malignant cells, with pronounced colocalization of high-NS malignant cells and OLR1^+^ macrophages in ccRCC tissues (Figure 9a). Additionally, we used the SpaGene package to evaluate the spatial activity of the EDN1–EDNRA signaling pair, which was specifically activated in regions where high-NS malignant cells and OLR1^+^ macrophages were co-localized (Figure 9b,c). Further analysis of the scRNA-seq dataset revealed that the proportion of OLR1^+^ macrophages was positively correlated with the abundance of high-NS malignant cells (*ρ* = 0.47, *p* = 0.033), but not with low-NS malignant cells (*ρ* = –0.28, *p* = 0.22) (Figure 9d). These findings suggest that the EDN1–EDNRA axis may contribute to the formation of an immunosuppressive microenvironment supporting ccRCC progression. Moreover, analysis of cell-type proportions from the scRNA-seq dataset in the TCGA-ccRCC cohort showed that patients with higher infiltration of OLR1^+^ macrophages had significantly worse OS (*p* = 0.0089, log-rank test) and PFS (*p* = 0.032, log-rank test) compared to those with lower macrophage infiltration (Figure 9e). These results underscore the clinical significance of OLR1^+^ macrophage infiltration in primary ccRCC. To further investigate the interaction between high-NS malignant cells and OLR1^+^ macrophages, we generated *Ets1*-overexpressing high-NS malignant cells via lentiviral transduction of Renca cells. Stable overexpression was confirmed by qRT-PCR (Figure 9f). Syngeneic transplantation models were established by subcutaneous injection of Renca LV-control (LV-Con) and LV-Ets1 cells into BALB/c mice (Figure 9g). Tumors derived from LV-Ets1 cells demonstrated accelerated growth and significantly increased tumor weight compared to LV-Con controls (Figures 9h, 9i, and 9j). Flow cytometry analysis revealed that *Ets1* overexpression markedly enhanced OLR1^+^ macrophage infiltration in ccRCC tumors (Figure 9k). Phenotypic profiling indicated that OLR1^+^ macrophages in LV-Ets1 tumors exhibited predominantly immunosuppressive features, in contrast to proinflammatory phenotypes observed in the LV-Con group (Figure 9l,m).

Our findings demonstrate that hypoxia-induced ETS1 activation drives NS via the MYC–NPM1/DDX17 axis. High-NS malignant cells collaborate to establish an immunosuppressive microenvironment through crosstalk with OLR1*^+^* macrophages, thereby promoting ccRCC progression. These results highlight the pivotal role of NS in shaping the immunosuppressive landscape of ccRCC.

## 4. Discussion

In this study, we characterized ccRCC subtypes associated with NS. Our results revealed that elevated NS was significantly correlated with an immunosuppressive TME and poor prognosis. By integrating single-cell transcriptomics and preclinical models, we demonstrated that ccRCC tumor cells in hypoxic pathological microenvironments activated the TF ETS1, thereby inducing the NS response. The excessive accumulation of ribosomal proteins driven by heightened NS was closely associated with tumor cell proliferation, glycolytic reprogramming, and immune cell infiltration. As a key driver of malignancy in several cancers—including breast, colorectal, hepatocellular, and lung cancer—elevated ETS1 expression at the protein or mRNA level correlates with higher tumor grade, poor differentiation, and increased invasiveness [27]. The hypoxic microenvironment characteristic of solid tumors, resulting from rapid proliferation-induced oxygen depletion and aberrant vasculature, has been shown to play a critical role in ccRCC initiation, progression, and prognosis. Notably, ETS1, a hypoxia-responsive gene, markedly promotes ccRCC progression and is associated with unfavorable clinical outcomes [28]. Wang et al. reported that ETS1 regulates ccRCC proliferation, metastasis [29], and invasiveness, and that its downregulation suppresses malignant behavior. Qian et al. further showed that ETS1 activation contributes to acquired resistance to tyrosine kinase inhibitors (TKIs), highlighting its potential as a therapeutic target for improving TKI sensitivity [30]. Verschoor et al. demonstrated that ETS1-overexpressing ovarian cancer cells exhibit glycolysis dependency, supporting its role in regulating tumor metabolic reprogramming [31]. Together, these findings elucidate the molecular mechanism through which hypoxia-induced ETS1 activation drives NS, promoting autonomous proliferation and metabolic adaptation in ccRCC. Additionally, spatial transcriptomics and syngeneic mouse models confirmed the spatial coenrichment of ETS1-activated high-NS malignant cells with immunosuppressive OLR1^+^ macrophages. Given Visium's near-single-cell (not single-cell) resolution, we interpret the observed co-enrichment at the spot level and will validate it in an independent cohort using multiplex immunofluorescence (pan-CK, OLR1, and hypoxia/NS) with cell segmentation and spatial proximity metrics (e.g., nearest-neighbor distance and Ripley's *K*). This study provides a theoretical basis for precise ccRCC subtyping and supports the development of therapeutic strategies targeting the NS pathway.

The nucleolus, once considered merely a site for ribosomal RNA synthesis, is now recognized as a central hub for cellular stress responses and a key regulator of tumor biology [17]. Under stress conditions such as hypoxia, the nucleolus undergoes structural and functional alterations that activate a cascade of signaling pathways influencing cellular physiology and disease progression [16]. Our findings suggest that hypoxia-related signaling induces NS via the ETS1–MYC–NPM1/DDX17 axis, resulting in the abnormal accumulation of ribosomal proteins in ccRCC. Previous studies have shown that targeting NPM1 and DDX17 can effectively suppress tumor progression [32–34], and our data further support their potential as therapeutic targets.

The NS signaling pathway can be broadly categorized into p53-dependent and p53-independent mechanisms, with the latter remaining poorly characterized [35, 36]. We observed significantly reduced p53 pathway activity in tumor cells classified as high-NS, offering new insights into p53-independent NS mechanisms. However, the precise correlation requires further investigation. In line with the notion that NS can proceed via p53-independent routes, our data show reduced p53 pathway activity in high-NS malignant cells. This pattern suggests that hypoxia–ETS1–MYC/NPM1/DDX17–driven ribosome biogenesis may bypass or dampen canonical RP–MDM2–p53 checkpoints, thereby favoring proliferative and metabolic adaptation. Future work will stratify tumors by TP53 status and test whether pharmacologic modulation of ribosome biogenesis or NS regulators restores p53 signaling or exerts antitumor effects independently of p53. Notably, ribosome biogenesis emerged as a core process associated with the high-NS phenotype, and it is intimately linked to tumor cell proliferation, invasion, metastasis, and immune evasion [37, 38]. Elevated levels of the ribosomal protein RPL19 have been reported in subsets of patients with liver and prostate cancer and serve as a sensitive biomarker of cancer progression [39, 40]. Therefore, the NS phenotype defined in this study may represent a novel therapeutic target in ccRCC. Inhibition of ribosome biogenesis or suppression of aberrant ribonucleoprotein accumulation may impair tumor cell proliferation and invasion [37].

Our findings also suggest that high NS is strongly and positively correlated with an immunosuppressive TME, with OLR1^+^ macrophages enriched around high-NS malignant cells. This macrophage subpopulation has been identified as a TAM subset in multiple tumor types [41–43] and is closely associated with poor prognosis. Additionally, previous studies have indicated that OLR1 serves as an immune-related prognostic biomarker and may function as a predictive indicator for immunotherapy efficacy [44]. Therefore, targeting OLR1 may represent a potential therapeutic strategy for slowing ccRCC progression. Collectively, these findings may help explain our observation that high-NS markers possess cross-sectional prognostic relevance and suggest their broader clinical significance in other solid tumors.

This study has several limitations. Due to the limited availability of public multiomics datasets for ccRCC, our findings may not fully represent the heterogeneity of the ccRCC patient population. Although we performed preliminary validation of our integrative transcriptomic analyses through cellular and animal experiments, the direct mechanistic role of hypoxia-induced NS in reshaping the immune landscape of ccRCC requires further experimental elucidation. Future research should prioritize large-scale, multi-institutional studies to confirm these findings and enhance their generalizability. In addition, more in-depth investigations are warranted to delineate the functional roles of individual genes associated with ccRCC progression and adverse clinical outcomes. These efforts will support the development of precise therapeutic strategies tailored to specific molecular subtypes of ccRCC.

## Data Availability

The data that support the findings of this study can be obtained from the corresponding author upon reasonable request.

## Supporting Information

Supporting Information Figure S1: Identification of NS-related molecular subtypes in ccRCC. Figure S2: Single-cell analysis of the ccRCC samples. Figure S3: Single-cell analysis of the malignant cells. Figure S4: Single-cell analysis of the myeloid cells. Figure S5: The expression of EDN1-associated receptors and targets.
