# Supplementary material for: ETS1-Driven Nucleolar Stress Orchestrates OLR1+ Macrophage Crosstalk to Sustain Immunosuppressive Microenvironment in Clear Cell Renal Cell Carcinoma
Source: Hum Mutat. 2025 Oct 3;2025:8856239. doi: 10.1155/humu/8856239 (PMC12513783; doi:10.1155/humu/8856239)
Supplement: Supporting Information — Figure S1: Identification of NS-related molecular subtypes in ccRCC. Figure S2: Single-cell analysis of the ccRCC samples. Figure S3: Single-cell analysis of the malignant cells. Figure S4: Single-cell analysis of the myeloid cells. Figure S5: The expression of EDN1-associated receptors and targets. [file 8856239.f1.zip › Supplementary Material.pdf]

Supplementary Material

ETS1-driven nucleolar stress orchestrates OLR1^{+} macrophage crosstalk to sustain immunosuppressive microenvironment in clear cell renal cell carcinoma

Lei Xiao[1], Zicheng Zhang[1], Tong Li[1], Yuyin Jiang[1], Yuanxin Liu[1], Jia Wang[2*], Wei Tang[1*]

1. Department of Endocrinology, Geriatric Hospital of Nanjing Medical University, Nanjing, Jiangsu, 210024, China.
2. Hohai University Hospital, Nanjing, Jiangsu, 210017, China.

*Correspondence: Wei Tang, Department of Endocrinology, Geriatric Hospital of Nanjing Medical University, Nanjing, Jiangsu, 210024, China. (E-mail: drtangwei@njmu.edu.cn). Jia Wang, Hohai University Hospital, Nanjing, Jiangsu, 210017, China. (Email: wangjia6600@126.com).

The PDF file includes:

Supplemental Figures S1 to S5.

Table of contents:

Supplement figures:

Supplemental Figure S1. Identification of NS-related molecular subtypes in ccRCC.

Supplemental Figure S2. Single-cell analysis of the ccRCC samples.

Supplemental Figure S3. Single-cell analysis of the malignant cells.

Supplemental Figure S4. Single-cell analysis of the myeloid cells.

Supplemental Figure S5. The expression of EDN1-associated receptors and targets.

Supplemental figures and legends

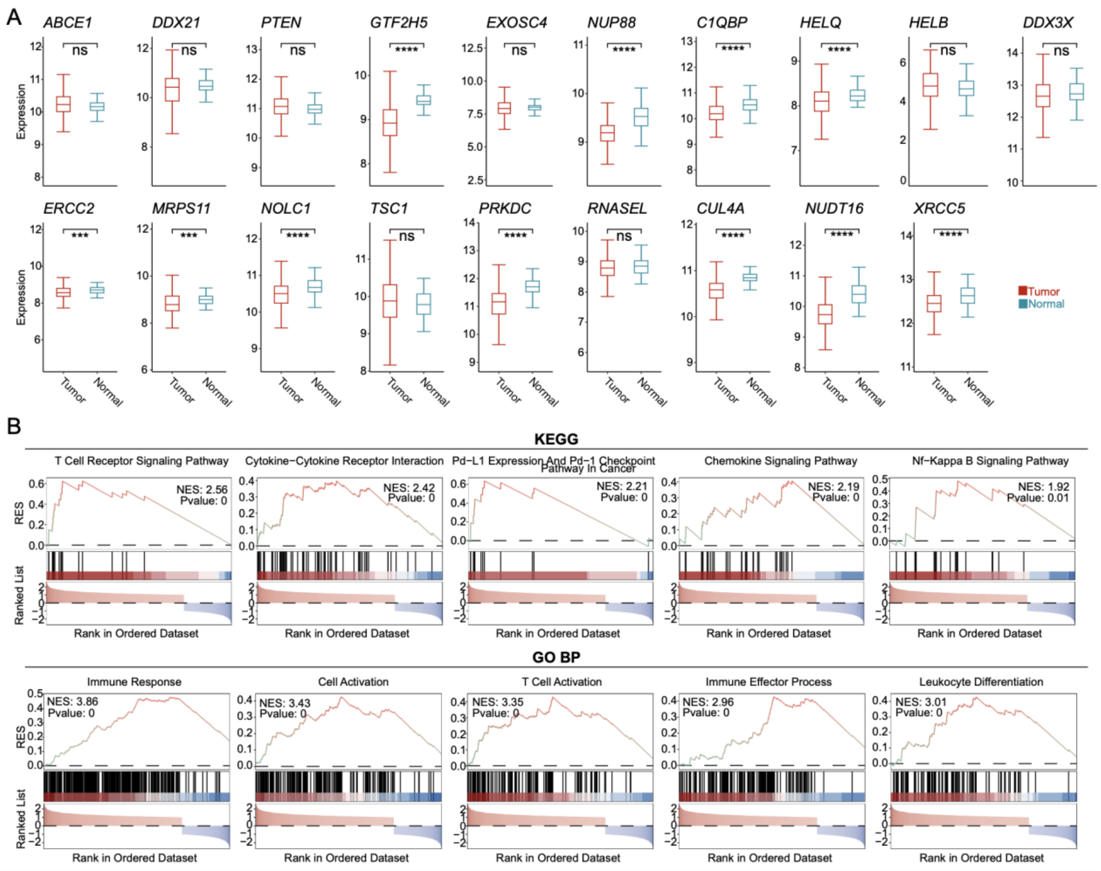

Supplemental Figure S1. Identification of NS-related molecular subtypes in ccRCC. (A) Box plots showing expression of other NS-associated genes (*ABCE1, DDX21, PTEN, GTF2H5, EXOSC4, NUP88, C1QBP, HELQ, HELB, DDX3X, ERCC2, MRPS11, NOLC1, TSC1, PRKDC, RNASEL, CUL4A, NUDT16,* and *XRCC5*). Different colors represent different sample types. B. Gene set enrichment analysis (GSEA) of KEGG and GO BP pathways between high- and low-NS. Ranking genes by fold change in expression between these two conditions.

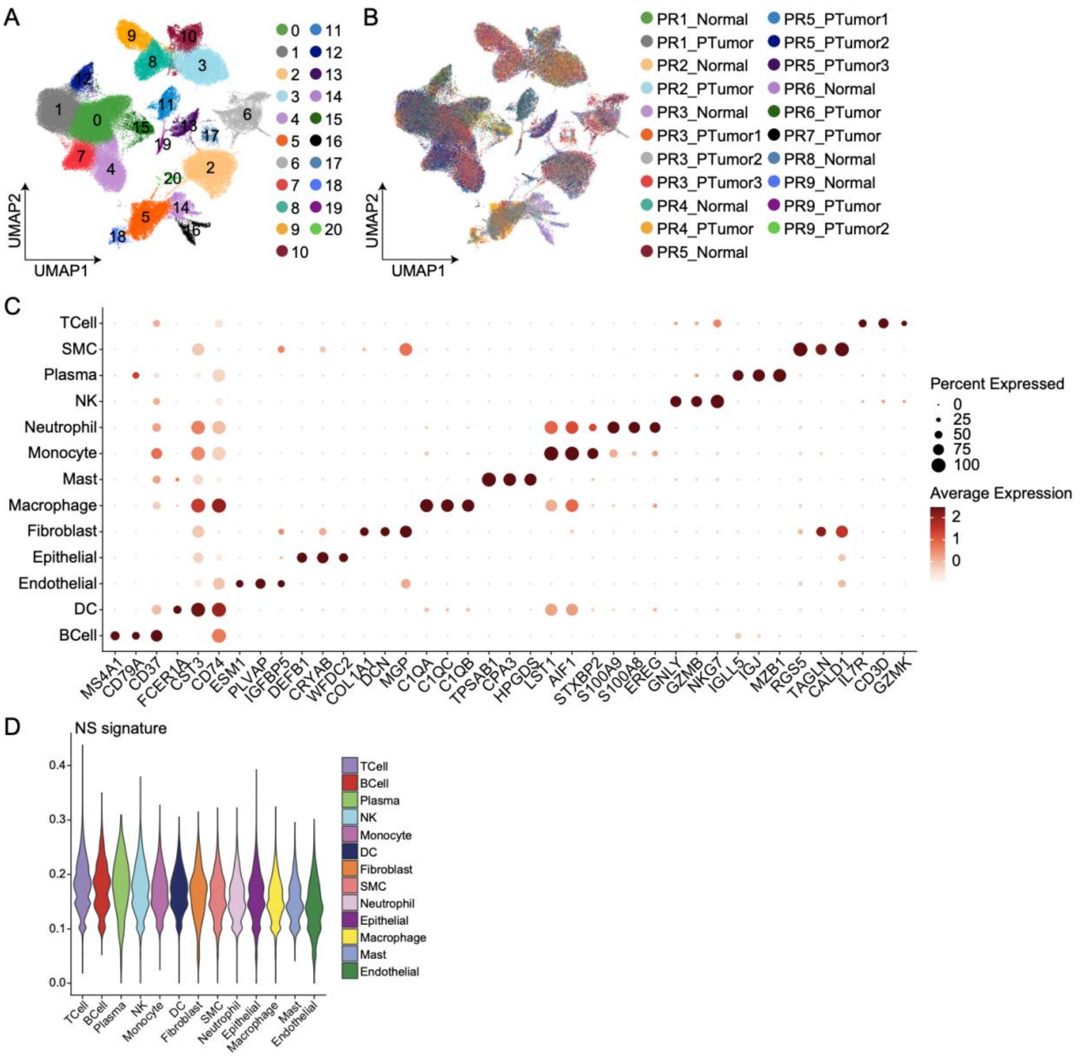

Supplemental Figure S2. Single-cell analysis of the ccRCC samples. (A) UMAP plot showing the clusters, with different colors representing different clusters. (B) UMAP plot showing the sample resources, with different colors representing samples. (C) Dot plot showing the expression of the top3 markers in each cell type. (D) The violin plots show the NS-associated signature in each cell type, with different colors representing different cell types.

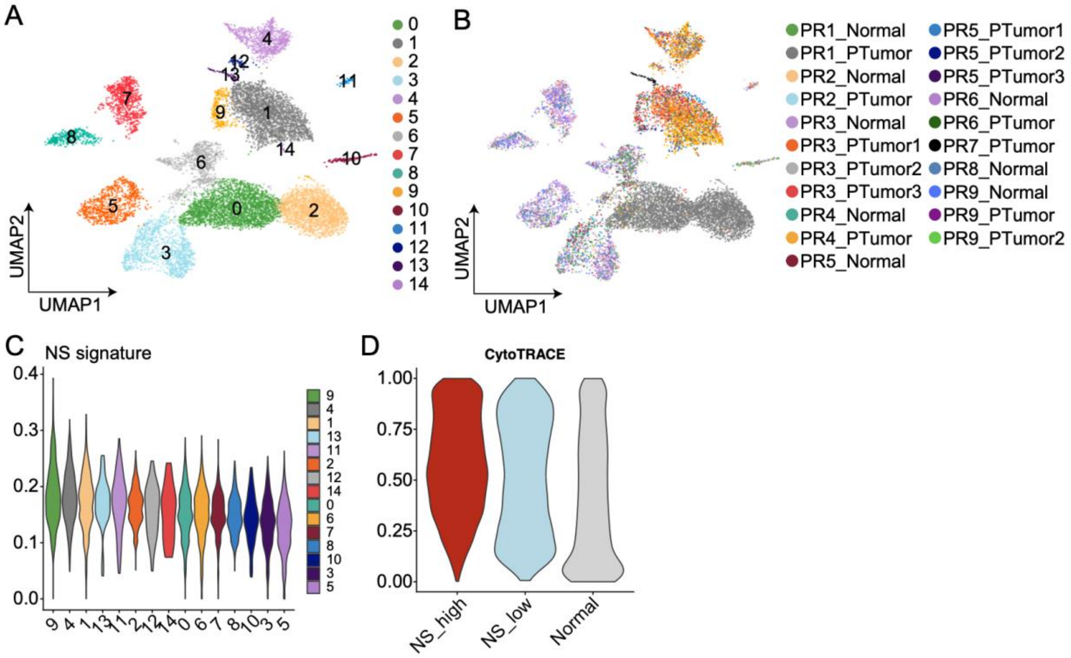

Supplemental Figure S3. Single-cell analysis of the malignant cells. (A) UMAP plot showing the clusters, with different colors representing different clusters. (B) UMAP plot showing the sample resources, with different colors representing samples. (C) The violin plots show the NS-associated signature in each cluster, with different colors representing different clusters. (D) The violin plots show the NS-associated signature in each malignant subtype, with different colors representing malignant subtypes.

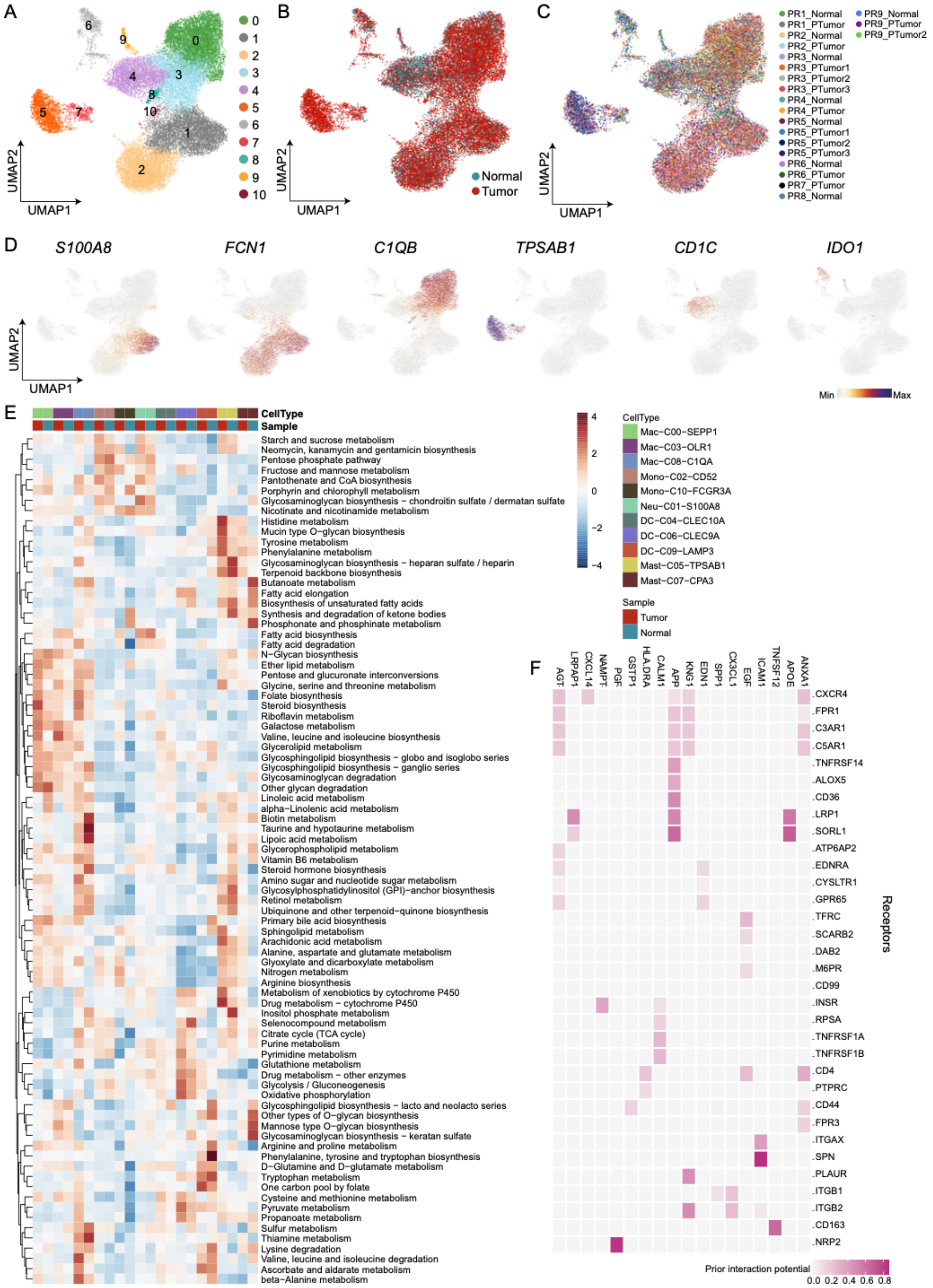

Supplemental Figure S4. Single-cell analysis of the myeloid cells. (A) UMAP plot showing the clusters, with different colors representing different clusters. (B) UMAP plot showing the distribution of sample types, with different colors representing sample types. (C) UMAP plot showing the distribution of

samples, with different colors representing samples. (D) UMAP plots showing the expression of *S100A8, FCN1, C1QB, TPSAB1, CD1C,* and *IDO1*. (E) Heatmap showing the activity of KEGG metabolism pathways. (F) ligand-receptor pairs are inferred to regulate the myeloid subpopulations by high-NS malignancy, according to NicheNet.

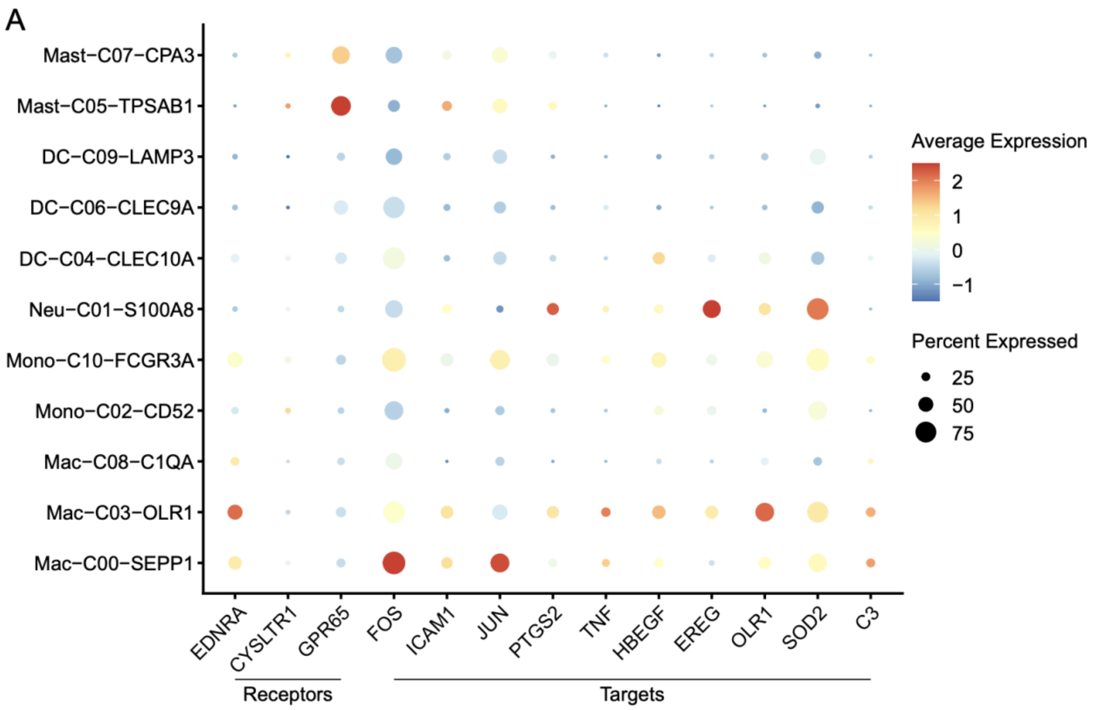

Supplemental Figure S5. The expression of EDN1-associated receptors and targets. (A) Dot plot showing the expression of EDN1-associated receptors and targets.
